# Robotic transanal total mesorectal excision: A new perspective for low rectal cancer treatment. A case series

**DOI:** 10.1016/j.ijscr.2019.07.034

**Published:** 2019-07-19

**Authors:** Igor Monsellato, Alessia Morello, Marta Prati, Giulio Argenio, Domenico Piscioneri, Luca Matteo Lenti, Fabio Priora

**Affiliations:** Division of General Surgery, Azienda Ospedaliera SS Antonio e Biagio e Cesare Arrigo, Alessandria, Italy

**Keywords:** Robotic transanal surgery, Rectal surgery, TME, Minimally-invasive surgery, Rectal cancer

## Abstract

**Background:**

Rectal cancer treatment is still a challenging frontier in general surgery, as there is no general agreement on which surgical approach is best for its management. Total mesorectal excision (TME), influenced the practical approach to rectal cancer, and brought a significant improvement on tumor recurrence and patients survival. Robotic transanal surgery is a newer approach to rectal dissection whose purpose is to overcome the limits of the traditional transabdominal approach, improving accuracy of distal dissection and preservation of hypogastric innervation. An increasing interest on this new technique has raised, thanks to the excellent pathological and acceptable short-term clinical outcomes reported.

**Materials and methods:**

Three consecutive cases of robotic transanal TME were prospectically performed between May 2017 and October 2017.

**Results:**

TME quality was Quirke 3 grade in all cases. Mean operative time was 530 min. None of the patients had intra-operatively or post-operatively complications.

**Conclusions:**

Robotic transanal TME is a very recent procedure. Acclaimed greatest advantage of robotic transanal TME is the facilitation of dissection with an in-line view, which translates in an improved surgical field exposure and visualization. Further investigations are needed to assure the actual value of robotic transanal approach.

## Highlights

• Robotic transanal surgery is a newer approach to rectal dissection.
• Results are promising in terms of mesorectal integrity and resection margins.
• Robotic system has many advantages for performing TME.

## 1 Introduction

Rectal cancer treatment is still a challenging frontier in general surgery, as there is no general agreement on which surgical approach is best for its management. A recent meta-analysis, indeed, included 14 unique RCTs and analyzed the rate of positive circumferential margin and the quality of mesorectal excision, concluding that the risk for achieving a noncomplete mesorectal excision is still significantly high in laparoscopic surgery [1]. Total mesorectal excision (TME), influenced the practical approach to rectal cancer, and brought a significant improvement on tumor recurrence and patients survival [2,3]. Introduction of robotics, has reached an important milestone to better short-term outcomes, reducing postoperative complications and morbidity [4]. Robotic TransAnal Surgery (RATS) is a newer approach to rectal dissection whose purpose is to overcome the limits of the traditional transabdominal approach, improving accuracy of distal dissection and preservation of hypogastric innervation [5]. Although there are few published cases in literature, an increasing interest on this new technique has raised, thanks to the excellent pathological and acceptable short-term clinical outcomes reported. Herein we describe our results on the first three consecutive cases of robotic transanal TME, prospectically included in a pilot study.

## 2 Materials and methods

Three consecutive cases of robotic transanal TME were prospectically performed between May 2017 and October 2017 in a community hospital. All intervention were performed by experts well-trained minimally invasive surgeons. Main comorbidities were COPD, diabetes and benign arterial hypertension (Table 1). Preoperative studies included colonoscopy with biopsy, complete CT-scan and pelvic MRI. Clinical stage was cT2cN + and cT3cN + in the last two cases. Neoadjuvant treatment was indicated, after multidisciplinary board, in all cases and consisted in long-course radiotherapy with a total dose of 50.4 Gy with boost in SIB, associated with oral capecitabine, according to the international guidelines. Restaging was carried out by CT-Scan and Pelvic MRI as usual and showed a complete lymph nodal response with no tumor response (ycT2N0) in the first case, a complete lymph nodal and partial complete tumor response (ycT2N0) in the second case and no lymph nodes and tumor response (ycT3N+) in the third case. An informed written consent was obtained from all the three patient for surgical procedure. The work has been reported in line with the PROCESS criteria [6].

**Table 1:** patients characteristics and preoperative and postoperative results and outcomes. ARF: acute renal failure. N: No; CRM: circumferential margin; BMI: body mass index; COPD: Chronic obstructive pulmonary disease.

	Case 1	Case 2	Case 3
Age	68	61	55
Sex	F	M	M
BMI	25	27	28
Comorbidities	Diabetes	COPD Benign arterial hypertension	–
Tumor distance from anal verge (cm)	6	4	3
cStage	T2N+	T3N+	T3N+
ycStage	T2N0	T2N0	T3N+
ypStage	T0N0	T2N0	T3N0
TME grade (Quirke)	3	3	3
CRM (mm)	>1	>1	>1
Distal margin	Clear	Clear	Clear
Blood loss	inconsistent		
Surgical technique	Robotic transanal first and then abdominal		Simultaneous laparoscopic abdominal phase and robotic transanal phase
Overall operative time (min)	550	600	440
Postoperative stay (days)	10	15	7
Intraoperative complications	N	N	N
Postoperative complications	N	N	N
Late complications	N	ARF (readmission)	N

### 2.1 Surgical technique

All patients underwent a complete mechanical bowel preparation with polyethylene glycol and received parenteral antibiotics prophylaxis prior to surgery. Patients were positioned in moderate Trendelemburg in dorsal lithotomy to facilitate perineal view and transanal access. The da Vinci Si Surgical System (Intuitive Surgical, Sunnyvale, California, USA) was used during the whole procedure. The first step of the procedure was the transanal approach, then the abdominal phase; in case #3 we used a simultaneous “combined approach”, laparoscopic transabdominal and robotic transanal accesses. A lone star retractor was positioned to expose the anal canal and to perform intersphyncteric resection. Afterwards, closure of the rectal lumen was performed above the dentate line with a purse-string suture.

**Fig. 1:**
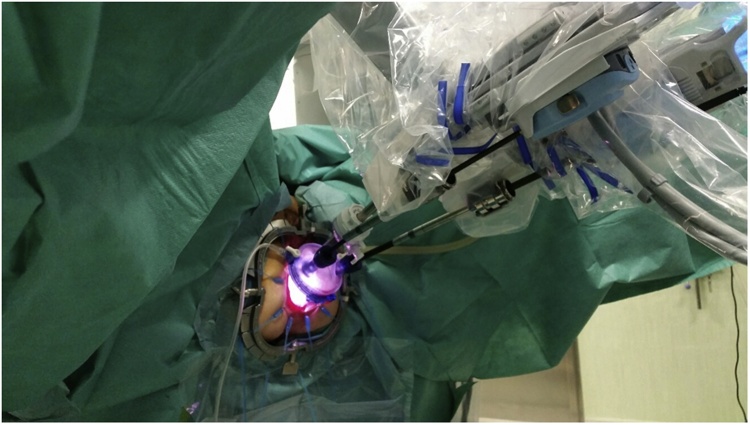
Patient and robotic instruments position.

**Fig. 2:**
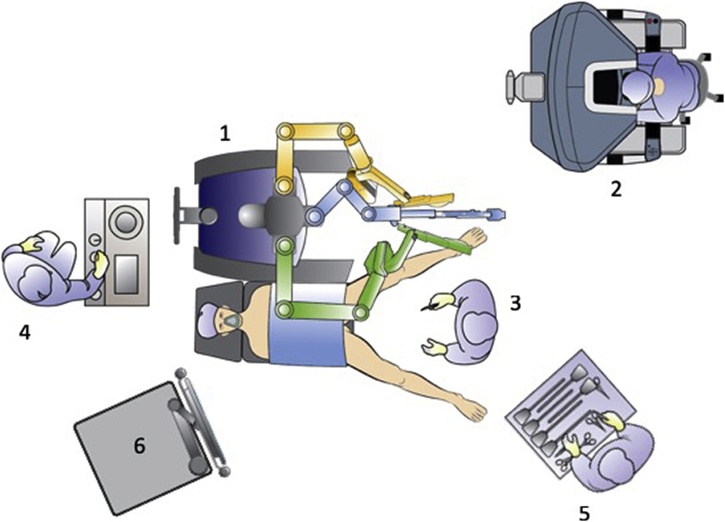
Operative Theater setup: 1 Robotic cart; 2 robotic console; 3 bedside assistant; 4 anesthesiologist; 5 scrub nurse; 6 robotic tower.

**Fig. 3:**
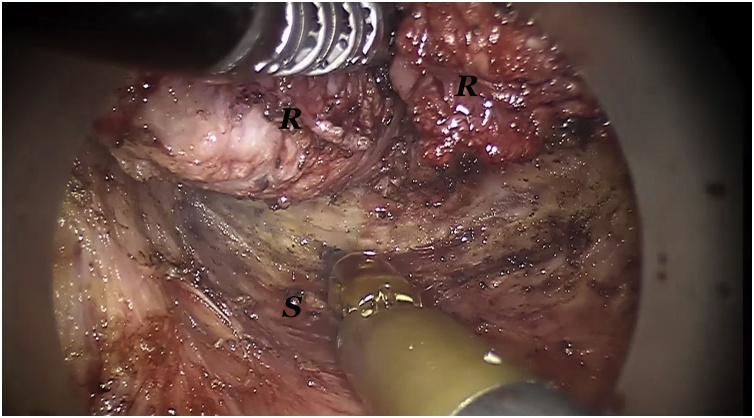
Intraoperative view: posterior dissection along the holy plane. R: mesorectum and rectum; S: posterior pelvic fascia. Mesorectum and rectum is retracted by the Maryland grasper.

**Fig. 4:**
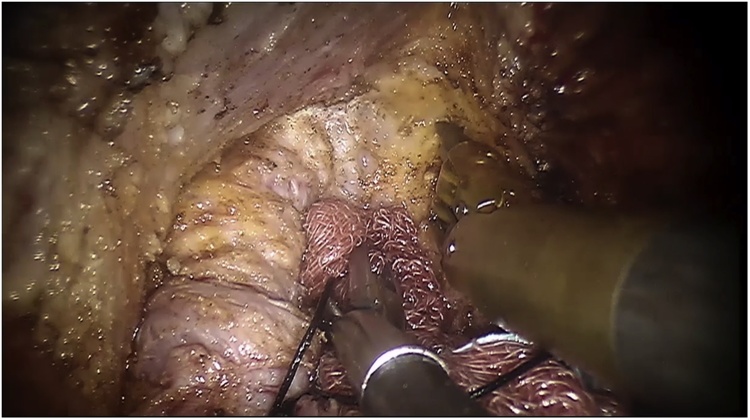
Intraoperative view: anterior dissection. A sponge is used by the assistant for rectal stump retraction. Console surgeon carries out the dissection with the spatula while retracts the anterior pelvis by the Maryland grasper (not in view).

**Fig. 5:**
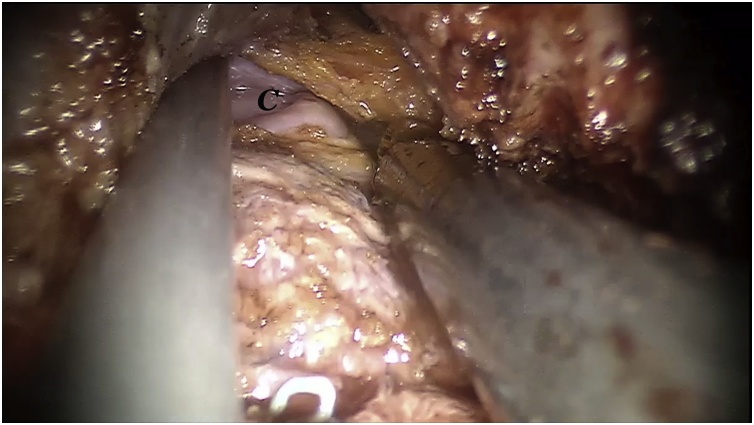
Intraoperative view: Anterior dissection. The peritoneal cavity has been reached and the peritoneal brim has been incised. C: abdominal cavity.

The single port-device GELPoint (Applied Medical Inc., Rancho Santa Margarita, CA, USA) was inserted transanally and a CO2 was inflated till reaching a 12 mmHg endoluminal pressure. A 12 mm laparoscopic trocar was inserted for the robotic endoscope, two GELPoint cannulas were introduced for the robotic instruments and a 5 mm laparoscopic standard trocar was also positioned below the optical access, for assistance (Fig. 1). The robot was docked from patient’s left side with arms #1 and #2 extended to the right (Fig. 2). We used a standard 0° robotic endoscope, a 5 mm Maryland type forceps on arm #2 and 8 mm Permanent Cautery Spatula on arm #1. The assistant used either the laparoscopic atraumatic forceps or suction instrument. Dissection began on the posterior side along the virtual plane between the presacral fascia and the anal canal with a caudocranial and a lateral direction. The rectosacral ligament was then incised and the virtual space between the mesorectal and the posterior pelvic fascia was gained (Fig. 3). The dissection continued along the anterior plane between the posterior face of the prostatic-seminal vesicles block (males)/vagina (females) and the anterior aspect of the rectum (Fig. 4). Once completed, the dissection ended up when the peritoneal brim was incised and the access to the abdominal cavity was achieved (Fig. 5). Afterwards, the abdominal phase started. Patient was first placed in anti-Trendelemburg position (5°), for splenic flexure takedown and then in Trendelemburg position (30°), with a slight right tilt (15°), for the vascular and left colon dissection phase. The robotic cart approached the operative table from patient’s left side with a 60° angle. Trocarts were placed in the usual manner: three 8 mm robotic trocars in right hypochondrium, right iliac fossa and left hypochondrium, respectively, and two 12 mm laparoscopic trocarts, one periumbilical for the endoscope and one in the right flank for assistance. A 30° robotic endoscope was used for the abdominal phase. The robotic fenestrated bipolar forceps was inserted on arm #2, the monopolar scissors on arm #1 and the Prograsp on arm #3. Splenic flexure takedown was performed by dissecting the gastrocolic ligament and the root of the mesocolon with a medial-to-lateral direction. Afterwards, patient was placed in Trendelemburg position, robotic arms #2 and #3 were switched for the vascular phase. The Prograsp was inserted on arm #2 and the fenestrated bipolar forceps on arm #3. The inferior mesenteric vein was ligated at the level of the inferior margin of the pancreas and the inferior mesenteric artery was ligated at its origin from the aorta. The abdominal phase was completed as the previous plane of distal dissection had been reached. The sigmoid colon and the complex rectum/anal canal were extracted through the anus and the resection of the specimen was then performed before indocyanine green test. A coloanal anastomosis and a loop ileostomy were fashioned as usual.

In case #3 the abdominal phase was performed laparoscopically and simultaneously to the perineal phase. Patient was placed in Trendelemburg position with a right tilt all along the intervention. Three 12 mm laparoscopic trocarts were placed in the right flank, periumbilical and in the right hypochondrium, respectively. Splenic flexure takedown and left colon dissection were carried out in the same manner as described above.

## 3 Results

RATS-TME was performed in three consecutive cases between May 2017 and October 2017. Mean age was 61 (55–68) years. Mean BMI was 26 (25–28). Tumors were located 6 cms, 4 cms and 3 cms from the anal verge and were preoperatively staged respectively as cT2N + and cT3N + the last two cases. All three patients underwent neoadjuvant treatment with chemoradiotherapy and, after restaging, surgical resection was made after 8 weeks. TME quality was Quirke 3 grade in all cases. Pathological stage was pT0N0, pT2N0, pT3N0, in patient #1, #2 and #3, respectively. Mean operative time was 530 min (440–600). A hand-sewn coloanal anastomosis was performed and a loop ileostomy was also fashioned in all three patients.

None of the patients had intra-operatively or post-operatively complications and were discharged on the tenth, fifteenth and seventh post-operative day, respectively. Patient #2 was discharged in 15^th^ POD because of bureaucratic issues related to relatives’ request for a continuity of care after discharge. Only one patient was readmitted to the ward eight days after being discharged, because of an acute renal failure, resolved by medication and intravenous hydration. After one-year follow up, two patients were disease-free and alive, one patient died for other causes not related to the tumor (cardiac failure). Loop ileostomy was closed after 2 months from the main surgery in all patients.

## 4 Discussion

Rectal cancer treatment is still a challenging frontier in general surgery, as there is no consensus on a standard therapeutic approach. Total mesorectal excision (TME) influenced practical approach to rectal cancer, introducing the new concept of” mesorectum” and the “sharp” technique of dissection that brought significant improvement in tumor recurrence and patients survival [1,2]. Surgical approach on distal rectum cancer can underlying issues to be addressed, because of several factors such as obesity, short tumor distance from anal verge, bulky tumors, and narrow pelvis, even if performed by a laparoscopic approach. Moreover, lack of adequate retraction and field of vision and misidentification of the distal resection margin are technical pitfalls of a transabdominal approach in low rectal cancer treatment [7]. Despite the development of the laparoscopic surgery, rate of complications, completeness of the resection margins (88 and 92 percent in the laparoscopic surgery group and in the open surgery group respectively), morbidity (40 vs 37 percent) and mortality (1 vs 2 percent) remain similar for both laparoscopic and open technique [7]. Furthermore a 21–29 percent of laparoscopic resections is converted to open resection, because of tumour fixity and adhesions, obesity, anatomic issues, poor exposure and uncertainty of tumour clearance, tumor inaccessibility, vessel injury, radiation fibrosis [[7], [8], [9], [10]]. These poor outcomes have led to a new impetus to the development of different technical options to reduce positive margin rate and improve oncological safety.

TAMIS (TransAnal Minimally Invasive Surgery) was first published in 2010 by Atallah et al. and included 50 patients (25 benign neoplasms, 23 malignant lesions, and 2 neuroendocrine tumors), in which it was used for tumor local excision [11]. In 2013, the same authors published their first case report of a robotic-assisted transanal surgery for TME (RATS-TME) to treat a cT3N1 cancer of the distal rectum, located 4 cms proximal to the anal verge. On pathologic exam, specimen margins were tumor-free, clear CRM, and TME was classified as Quirke grade 2. Overall operative time was 380 min. In 2014, Atallah et al. published a pilot study including three patients in which RATS was performed. Patient were positioned in moderate Trendelemburg position in dorsal lithotomy and robotic cart approached the table from patients’ right side. Mean operative time was 376 min. In each case, the distal and circumferential resection margins were free of tumor, with the closest distal margin of 1 cm and Quirke grade 2 and 3, in two and one case, respectively [5].

In our experience, RATS-TME was performed also in three consecutive cases. In two cases authors first performed the transanal approach and then the abdominal phase. In the last case authors performed simultaneously (two surgical equipes) the abdominal and the robotic perineal phases. Transabdominal approach was carried out laparoscopically, easily and with no issues related to the presence of the #2 robotic arm over the abdominal field, and flexure takedown, indeed, was conducted fluently by the so-called “upper approach”. We used a 5 mm Maryland grasper and a 8 mm spatula: we encourage the use of robotic spatula because of its flat shape that it seemed to offer some advantages in conducting a round and sharp dissection along the “holy plane”. Maryland grasper allowed a precise and low-trauma handling and retraction of the rectal stump, thanks to its curved shape and the endowrist. We also agree with Atallah et al. in using at least one 5 mm robotic instrument to avoid inside conflicts [5]. In the second case we had difficulties in positioning trocars in the correct place of the GELPoint, and this caused some issues in using the spatula and prolonged operative time. Trocarts position, usually resemble an inverted V-shape, with the optical trocart at the apex; cannula for spatula was positioned in case #2 slight upper than usual and even after reallocation, it still caused internal conflict with the endoscope. Mean operative time was 530 min: we assume that it was related not only to some issues in placing the two operative trocarts through the GELPoint in case #2 but also to the learning curve, even though all surgeons involved in all cases are well-trained and skilled experts in transabdominal robotic and laparoscopic surgery. Only in the first case we had some issues with bellowing, that we resolved as suggested by Atallah et al., leaving the lure-lock for smoke evacuation closed, and by the use of laparoscopic suction device via a fourth port. CO2 pressure was set at 12 mmHg, and in the last two cases was stable [5]. TME was carried out easily and pathological specimens showed a complete mesorectum and disease-free resection margins. Even though main limitation of our study is the number of patients, we can assume that our results could corroborate the promising results of transanal robotic surgery, as reported also by Vignali et al. [12].

Laparoscopic TaTME is nowadays a widespread procedure that has been introduced in clinical practice in order to improve the quality of surgery not only for the management of low but also mid or even high rectal cancer [13]. On the other hand, some authors have also reported specific intraoperative complications observed during TATME, such as urethral or bladder injury, and rectal or vaginal perforation and considered the end of the learning curve after the first 20 cases [[14], [15], [16]].

Robotic system has many advantages for performing TME thanks to its several aspects: better vision with a magnified 3D view and a high definition, a more stable platform, a self-controllable camera, instruments with more degrees of freedom and without tremor, improved opportunity to control unexpected bleeding and better ergonomics, that could reduce the time-period and number of cases to complete the learning curve in TaTME, and intraoperative complications allowing better visualization of the correct anatomical plane of dissection [17].

A recent review has established that there is no significant difference between laparoscopic and robotic TAMIS in terms of peri-operative parameters and 30 day post-operative complications other than total direct cost, even though L-TAMIS is technically and ergonomically demanding, thus proposing R-TAMIS for a more aggressive approach with respect to resection of rectal neoplasms [18]. Increased cost is still considered an issue for robotic surgery: Atallah et al. robotic transanal surgery reported an increased cost per-case of $1500, including the cost for GELPoint platform [5].

More recently, however, a structured cost analysis of robotic TME resection for rectal cancer has demonstrated a significant reduction of costs with increasing surgeon's experience [19]. The introduction of the da Vinci Xi robotic platform (Intuitive Surgical, Sunnyvale, CA, USA) and the search for a simultaneous “easy” approach for rectal cancer is leading to new concepts of operation as the simultaneous robotic synchronous approach [20].

## 5 Conclusions

RATS-TME is a very recent procedure. Few reports are still available to draw final conclusions, but preliminary results have shown that is feasible and safe with good technical and oncological results. Robotic assistance confirmed its advantages, which are enhanced performing this type of procedure (endowrist, scaled motion, magnified and 3D vision). Acclaimed greatest advantage of RATS-TME is the facilitation of dissection with an in-line view, which translates in an improved surgical field exposure and visualization. Further investigations are needed to assure the actual value of robotic transanal approach.

## Sources of funding

No funding were used for this research.

## Ethical approval

The current study has been exempt from ethical approval by my Institution.

## Consent

Written informed consent was obtained from the patients for publication of this case report and accompanying images. A copy of the written consent is available for review by the Editor-in-Chief of this journal on request.

## Author contribution

I.M., A.M, M.P., G.A., D.P., L.L, F.P. have equally contributed to the analysis, interpretation and writing of the paper.

## Registration of research studies

Researchregistry 4892.

https://www.researchregistry.com/browse-the-registry#home/registrationdetails/5ce3e2d40cf6af000613269c/.

## Guarantor

Igor Monsellato Md PhD.

## Provenance and peer review

Not commissioned, externally peer-reviewed.

## Declaration of Competing Interest

Authors declare to have no conflicts of interest.
